# The Causes of Exudation in Inflammation

**Published:** 1864-09

**Authors:** 


### Selections.

THE CAUSES OF EXUDATION IN INFLAMMATION.—*By Mr. Edward Greely Loring, Jr. An Essay to whih one of the Prizes of the Boylston Medical Society was Awarded.**

THE object of the following Essay is to prove how far in an inflammatory process the exudation is the result of the inflammation, not a part of it, and how far it is dependent on the saline ingredients of the economy.

The following statements were taken from one of Dr. White's lectures at the Hospital on the clinical examination of the urine. "The chloride of sodium has a great effect in rendering substances in the blood soluble. Blood contains in every 1000 parts 4.2 parts of chloride of sodium, serum 4.6. Effusion in pleurisy, 7.5. Ten and one half grammes pass out of the system by the kidneys in twenty-four hours.

* A prize awarded to under-graduates of Harvard Medical School.

In acute diseases, where there is effusion into serous cavities, joints or tissues, the chlorides are diminished in the urine. In pneumonia the chlorides are very much and very rapidly diminished—sometimes totally in twenty-four hours. When reabsorption takes place, the chlorides reappear in the urine. In inflammation of the liver, in peritonitis, and in cholera, there is often a total absence of the chlorides in the urine. If the chlorides reappear, convalescence takes place. In typhoid fever the chlorides are diminished, though not so much, nor so rapidly, as in the above-mentioned disease. In intermittent fever they are diminished on the fever day. The urine only contains the surplus of what is not wanted by the blood. The kidneys refuse to eliminate the chlorides where there is inflammation with effusion. If, when the kidneys will not eliminate it, salt be passed in quantities into the system, it will then be eliminated through the intestinal canal, but not until the blood is saturated. When the chlorides are diminished in the urine, the other saline ingredients are also diminished, though in a less degree. In rheumatism the chlorides in the urine are but slightly diminished, though when pericarditis is associated with it, they are diminished to a considerable extent."

It will be seen by the above statements, that, in all diseases characterized by an exudation, the chlorides (the chief of which by far is the chloride of sodium) are diminished in the urine, and, that the more inflammatory the disease is, the greater is the decrease, and that as they decrease in the urine, so they increase in the exudation, since the serum of the blood contains but 4.6 parts in 1000, while the effusion resulting from an inflammatory disease, where the chlorides are diminished in the urine, contains 7.5 parts. Now as the diminution of the chlorides in the urine is accompanied by their increase in the exudation, and, as exudation is often the result of inflammation, the question arises, what relation their disappearance in one place bears to their appearance in another, and to what extent, if any, does their diminution on the one hand, and their increase on the other, affect the process of inflammation, or at least that part of it which relates to effusion?

Without stopping to speculate on the vexed question of what inflammation is, or what its causes are, let us look at some of its effects after the process is once set in motion—the two most prominent of which effects are determination with increased flow of blood towards a certain spot, followed by a stagnation of blood and an exudation at that spot.

The exudation is the result of endosmosis and exosmosis. Certain laws govern this process, among which are the following:—

1. A non-saline or watery fluid will pass through a membrane towards a saline one, faster than the saline one will pass to it, and the rapidity with which the watery fluid will pass to the saline is in proportion to their different consistency.

2. The quicker the current of a liquid on one side of a membrane the greater the attraction, consequently, the greater the endosmosis into that liquid.

3. The activity of endosmosis and exosmosis is in direct proportion to the extent of surface over which the two liquids come in contact with the intervening membrane.

4. Pressure exerted on fluids favors their passage through a membrane.

5. Affinity and the desire which a fluid has to maintain its integrity favors the process of endosmosis.

In inflammation there is a great increase in the metamorphosis of tissue and an increased flow of blood to the part for the purpose of repairing in some way the waste, occasioned by the process which we call inflammation. Now the greater the inflammation, the more the waste, and the greater the determination of blood to repair that waste. The process by which tissues are repaired is the same as that by which they are nourished, in disease as in health, viz., that of transudation, known as the process of endosmosis and exosmosis. It is sometimes thought that the exudation which characterizes inflammation is due to the fact that the blood, in passing through the inflamed portion, gives up its watery parts, and then in its altered condition passes on, while its place is supplied by fresh blood, which, in its turn, undergoes the same process. Now if this were true, the more rapid the current of blood the greater the exudation, which is diametrically opposed to the law proved by Matteuci, that the force of transudation was *into* the current, not *from* it, and that this force was in proportion to its rapidity. Again, if this were the case, the greatest amount of exudation would be at the highest point of the inflammation. But every practising physician knows that the force of the inflammation and the rapidity of the current begin to subside as the exudation appears. This is not only true chemically, but is also in accordance with physical laws. Henle says: "As a physical consequence of the dilation of the vessels, there takes place a retarded flow of the blood. This together with the relaxation and dilation of the vessels, favors the exudation of the serum, the consequence of which is that the plasma of the blood becomes inspissated by a preponderance of the protein matters over the salts." This is quoted here simply to prove that the flow is retarded, not to answer the question what occasions the "stasis," which, according to Mr. C. Hanfield Jones, "is the great unsolved problem of inflammation." But more of this hereafter.

Taking into consideration all the above facts and laws, rational and physical, let us boldly put forth the theory that the exudation is the result of inflammation, not a part of it; and it is dependent on the saline ingredients of the economy, "of which the chloride of sodium is rather more abundant in the blood than all the rest together," and then let us see how far our premises will support our conclusions.

Suppose active inflammation to exist, and with it, as a matter of course, an increase in the metamorphosis of the tissue, and that with this we also have an increased flow of blood towards the spot, caused, it is fair to suppose, by the thirst which the tissue has to repair the waste occasioned by the morbid action. This, it is true, is theoretical, but as long as the cause or causes of inflammation are enveloped in mystery, we have a right to indulge in a theory which is based on common sense, till a fact demonstrates it to be untrue. Is it not fair to suppose that the sensation which we call hunger, and that which we call thirst, are but the expression of the desire which the tissues have for that wherewith to replace what has been expended? Can not inflammation be supposed to hear the same relation to the natural process of nutrition that a morbid craving does to a natural appetite? In support of this theory, Mr. Simon, in his "Lectures on Pathology," remarks, "altogether, we may take it as an established certainty, that the first change which occurs in an inflamed or *overgrowing* part, and which leads to its becoming loaded with blood, is not a reflex change operated through the nerves, but is a direct change by the living molecular structure of the part on the blood which traverses it, or on the vessels which convey the blood." It is to be remembered that the increased and more rapid flow of the blood through the vessels are things which, by the aid of the microscope, we can see—but the molecular movements of nutrition and secretion, which we believe to influence and modify the circulation through a part, we can not see. They are, however, as real, as potent; but they are, except in their results, invisible.

Suppose, then, this active metamorphosis to be going on. and a very much larger amount of nutritive material to be consumed than that required in an ordinary state of health, might it not be possible, nay probable, of a plasma composed of watery, organic and inorganic compounds, that the liquid and easily decomposible organic matter should be more rapidly consumed than the inorganic or saline material, and in this manner might there not result an accumulation of the latter in the surrounding tissues and membranes? Or, even stronger, might not the tissues, influenced by the morbid action, have a direct desire for particular ingredients of the blood over the others, in which case we should expect to find a diminished amount of this particular ingredient in the blood, when affected by the inflammatory action, than when in a state of health? Now, according to Jones and Sieveking, "in violent inflammation the salts are much diminished," and according to Henle, through exudation of the watery and saline parts, the plasma of the blood becomes inspissated by a preponderance of protein matter over the salts.

In the blood we have seen that the chloride of sodium is more abundant than all the other saline ingredients put together, and we should naturally suppose, even had it not been proved, that its abstraction from the blood would be followed by grave results.

In case of inflammation with exudation, the "kidneys do not eliminate the chlorides." What becomes, then, of all the chlorides introduced into the system, especially the chloride of sodium, which is largely taken into the economy with whatever we eat, drink or breathe? As the kidneys do not eliminate it, we should expect to find the blood charged with it; but in inflammation the blood contains less than in health, when the kidneys are actively drawing it from the blood. If the kidneys do not eliminate, and the blood contains less than its normal amount, what becomes of it? The excess must exist somewhere, and it does exist in the exudation, where it is found in the proportion of 7.5 to 4.6 parts in 1000 of the healthy serum of the blood. But it is proved that when the kidneys refuse to eliminate the chlorides, they are eliminated by the intestinal canal; but this only happens, according to Dr. White, when the blood is saturated with them, and as in inflammation the blood is not only not saturated, but even contains less than its usual amount, this elimination can not take place.

We have supposed above that an inflammation existed, that there was an increased flow of blood towards a particular spot, that there was an increased metamorphosis of tissue, that there was an accumulation of the saline ingredients in the neighboring tissue. To support this last statement, which it is our object to prove, we have the following facts:—

1. Absence of the chlorides from the urine.
2. Decrease of them in the blood.
3. Increase of them in the exudation.

Let us now see how this increase in the exudation takes place, and to what it is owing.

To do this we must recapitulate, and imagine, as before, that the inflammatory process has been set in motion. The blood now rushes towards the inflamed part, forcibly drawn thither by the desire which the tissues have to supply the waste by new material. Arrived at the spot, it delivers to the tissues what they need. But the desire being greater than the supply can satisfy, fresh blood is attracted to the spot, and, this process continuing, a current is established, and the greater the desire, or, in other words, the greater the inflammation, the quicker the current. But it has been stated that the quicker the current is, the less the exosmosis, and consequently, the less the material passed to the tissues. This would be true were it not for the fact, that it is the affinity which the tissues on one side of the membrane have for what the blood contains on the other that causes the current; and it is easy to see that attraction towards a particular spot might cause a current towards that spot, with transudation *outward at that spot*, while in other places where the morbid action did not exist, the attraction would be *into* the current and inproportion to its rapidity. So it is with inflammation. What it furnishes to one place it abstracts from another, and so drains the system.

Therefore, suppose the circulation, and at the same time the transudation of the plasma to be rapid—since we have seen that these two conditions are not necessarily incompatible—a great amount of new material is then rapidly delivered to the tissues and is rapidly consumed, and the consumption of the protein compounds being greater than that of the saline from their very nature, it follows that the neighboring tissues will become incorporated with the salts, and this consumption of the organic and accumulation of the inorganic will go on as long as the morbid action lasts. But when this desire on the part of the tissues ceases, a new condition of affairs takes place. The rapidity of the current, which had been occasioned by the "attraction," diminishes as its cause becomes less, and the current would again regain its natural velocity, were it not that a new set of phenomena, purely physical, now occur. The condition is this:—Suppose the walls of the capillaries to represent, as they do, a membrane through which the process of endosmosis and exosmosis takes place. On one side of the membrane is the tissue incorporated with the salts, or a saline liquid, while on the other is the blood, or a watery liquid, and more so than usual, for, as Lehmann states, "in the beginning of most diseases, especially the acute ones, the blood is found more watery than usual." Now what takes place? In obedience to the law, the watery fluid passes to the saline, and the amount and velocity with which the former will pass to the latter are in proportion to the amount and density of the latter. What is the result? Exudation or infiltration on the one side, while the blood on the other, deprived of its watery and saline ingredients—the latter of which, according to Dalton, play so important a part in rendering the albumen soluble and maintaining the integrity of the globules—becomes thick and inspissated, and, the protein material predominating over the watery and saline, the fluid, from its very nature, becomes unfit for circulation, and stasis occurs; and the inflammatory action continuing, disintegration of the vessels and tissues follows. Can not this *questio vexata*, this matter of stasis, which so many anatomists have tried so hard to prove was the result of some invisible, vital force, be explained as the simple result of the above physical laws and their action?

In speaking of this matter of stasis, Mr. Wharton James says:—"The stagnation commences in the capillaries, and extends from them to the veins on the one hand and the arteries on the other;" and Mr. Hanfield Jones, writing on the same subject, says:—"We have seen the blood stagnant in the capillaries, while it was moving on steadily through an adjacent artery and vein. This points to the capillaries as the part where the arrest commences," and this is precisely where the exosmosis takes place.

We now have, as the result of the inflammatory action, a stoppage of the circulation at the spot and a thickened and altered blood, which the *vis a tergo* is unable to force through the vessels, on one side of the walls of the capillaries, the effusion on the other. With this we also have decrease of saline compounds in the blood—especially the chloride of sodium—and decrease and even absence of the chlorides in the urine. If the inflammatory action continues, the tissues will undergo further changes until degeneration of them and the surrounding parts ensues. But if the morbid process ceases, a different result follows. Strictly speaking, there can be but one end to inflammation—that which is called resolution, in which the diseased action ceases to advance, and then recedes by the same steps by which it arrived at the condition of stasis. The inflammatory process ceasing, the "craving" which the tissues had ceases also, and the attraction no longer existing, the desire on the part of the blood to regain its former consistency and to maintain its integrity causes the transudation to be into the capillaries, not from them, in conformity with, if not in obedience to the physical law that the lighter liquid passes to the heavier in proportion to the density of the latter, the affinity between the two liquids being in proportion to their difference. The force of the *vis a tergo* is now sufficient to propel the fluid through the capillaries, and circulation is re-established. These phenomena correspond to the subsidence of the general symptoms.

The redness and the pain become less, the temperature is lowered, and the swelling (caused, according to Rokitansky, not by the congestion of the vessels, but by the effusion) subsides. This is resolution, or reabsorption. But it is not water alone that the blood stands in need of; it wants also the salts which have been exhausted from it, to render its protein compounds soluble and to maintain the integrity of its globules. So as reabsorption takes place, not only does the blood regain its normal amount of saline ingredients, but these also *reappear in the urine,* in which they were either diminished or totally absent.

Now as we have seen, while the inflammatory action is at its height, there is a minimum of the saline ingredients of the blood, a minimum or absence of them in the urine, and a maximum in the effusion; and as the effusion disappears so do they reappear both in the blood and in the urine, it is fair to conclude, then, that the increased quantity in the effusion was obtained at the expense of that which previously existed in the blood and of that which would have been eliminated by the kidneys. This would follow rationally, had it not been *proved* by actual analysis that the effusion did contain more saline material than the serum of the blood from which the effusion is elaborated.

Taking all the above facts into consideration, we may at least have some reason for thinking that the exudation is not a part of the inflammatory process, but is rather the result of the morbid action depending on or occasioned by the saline ingredients of the blood, of which the chlorides play by far the most important part, and that the effusion is rather a physical than a vital process. Let us see, now, if this view will conform with the phenomena presented by actual disease.

We will begin with pleurisy. We select this affection because in other diseases the chemical examination of inflammatory products is very difficult, partly in consequence of the impossibility of procuring more than very small quantities, partly because they can be obtained so seldom in a pure and unmixed state. But the exudation of pleurisy, from the nature of its receptacle, approaches nearer to this condition of purity than would other exudations and infiltrations, where they are mingled with a variety of tissues by the changes in which they themselves would be apt to suffer change.

Case I.—A man is attacked with pleurisy, and goes through all the regular phases of the disease. During the attack the chlorides are diminished or disappear in the urine. Exudation takes place, accompanied with an amelioration in the inflammatory symptoms. The exudation is absorbed, and the chlorides reappear in the urine. Or, again, a man is attack with pleurisy. The chlorides have disappeared from the urine. The man is tapped, and the effusion is found to contain in every 1000 parts 7.5 parts of chloride of sodium alone, while the blood contains normally only 4.2 parts: the whole amount of fixed salts being, according to Simon, in fluid obtained by paracentesis thoracis, 9-5 parts in 1000.

Case II.—Pneumonia. A man has an undoubted attack of pneumonia. The chlorides disappear entirely from the urine. Convalescence, with reabsorption, takes place, and the chlorides reappear in the urine—or the man dies in the height of the disease. The chlorides were absent in the urine. At the autopsy, a general infiltration is found to have taken place into the tissue of the lung, and the salts in the blood, as in all violent inflammation, are found to be much diminished, while they are found in greater quantity in the sputa.

"In malignant cholera the excessive drains tell most on the fluid part of the blood, and hence that remaining in the vessels is thick and tar-like; hence, also, the extraordinary though temporary effect of injecting *saline solutions*, which return to the blood the material effused from it and revive all the functions that were well nigh extinct. Doubtless if the intestinal discharges could be arrested the effects would be permanent, but as it is, their effect is soon exhausted." If we, with the Germans, consider cholera to be an inflammatory disease, and in this the same metamorphosis of tissue to have taken place, the same consumption of the organic and deposit of the saline materials to have occurred as in other inflammations, we can easily understand why the exudation, occurring as it does, over such an extent of membrane, should be so excessive, and the drain on the system so great, the blood so thick and tar-like.

Many more instances of the same effect, taking place in other than the above mentioned diseases, might be brought forward here, such as in peritonitis, in meningitis, and in pericarditis, &c. But the limits of this article will not permit their insertion here.

But it may be said that rheumatism is an inflammatory disease, and that the same general phenomena must characterize this as other inflammatory processes; that there would be the same metamorphosis of tissue, the same preponderance of saline material, and if there were, there would also be, according to the physical law laid down, an exudation, and if this exudation did take place there should be a decrease of the chlorides in the urine. Now the exudation in rheumatism is comparatively scanty, and although there is some diminution in the salts in the urine, they are not nearly so much diminished in this as in other inflammatory diseases. This seems at first sight to be at variance with the views brought forward, but in reality is confirmatory of them, as we shall see if we consider that rheumatism generally attacks the joints, where the tissues, instead of being spongy or yielding, are compact and firmly bound by tendons and fasciæ, which by their pressure on the vessels greatly impede transudation. And we can easily understand that the inflammation, although confined in extent, might be exceedingly violent and painful, and the consumption of material at that spot great, while the supply of new material, from the pressure exerted on the vessels which should provide that part, would be limited; and the accumulation of the chlorides at this spot and their decrease in the urine, not so strongly marked here as they would be where the inflamed part was a serous sac, where the morbid action could have full sway, where the vessels ramify over a large extent of membrane, and where there is nothing to impede but everything to favor exosmosis—as, for example, in pleurisy, peritonitis, meningitis, &c., or in the spongy tissue of the lungs, or the yielding ones of the brain. But when we have pericarditis associated with and dependent on rheumatism, we have the inflammatory process extending over a comparatively large surface. In this case, where the process of waste and repair is not interfered with, the consumption of the organic mater untrammeled, the residue of the inorganic unaffected, and the exudation unimpeded by pressure on the ramifying vessels, we not only have the effusion, but also the accompanying diminution of the salts in the urine—as in the case of Albert Ford, now at the Mass. Gen. Hospital, in whose urine only the slightest trace of the chlorides was detected.

In the foregoing remarks perhaps sufficient distinction has not been drawn between exudation and simple transudation. Reference has been made solely to the exudation which is the result of the inflammatory process, not at all to that of simple transudation dependent on a weakened and relaxed state of the capillaries—for in the latter process the chlorides are not diminished in the urine, while according to Lehmann they are even increased in the blood. No allusion has been made to the exudation resulting from the inflammatory process known by the indefinite and erroneous name of "lymph," as this is considered by the writer to be a healthy, not a morbid action—a process of repair for the purpose of replacing the tissue destroyed, since under favorable circumstances this is capable of organization, though this, like the other tissues, should the inflammatory action continue, can be consumed or destroyed, and then the same result will follow here as that mentioned as occurring in the preceding remarks.

How far exudation is the result of inflammation and not a part of it; and how far it is dependent on the saline ingredients in the economy, we have by the above arguments and examples tried to prove. It would be useless to cite any more, for if there is any truth in the above remarks these will be enough to prove it; if there is no truth in them, then the fewer examples to prove an absurdity the better.—*Boston Medical and Surgical Journal.*

